# Flexible upscaling of laboratory PCR testing capacity at the Robert Koch Institute during the SARS-CoV-2 pandemic

**DOI:** 10.1186/s12985-023-02088-x

**Published:** 2023-07-05

**Authors:** Eva Krause, Janine Michel, Andreas Puyskens, Natalie Hofmann, Thomas Rinner, Barbara Biere, Brigitte G. Dorner, Martin Skiba, Lars Schaade, Andreas Nitsche

**Affiliations:** 1 Centre for Biological Threats and Special Pathogens, Unit Highly Pathogenic Viruses (ZBS 1), WHO Collaborating Centre for Emerging Infections and Biological Threats, WHO Reference Laboratory for SARS-CoV-2, Robert Koch Institute, Seestrasse 10, 13353 Berlin, Germany; 2 Department for Infectious Diseases, Unit Influenza and Other Respiratory Viruses (FG 17), Robert Koch Institute, Seestrasse 10, 13353 Berlin, Germany; 3 Centre for Biological Threats and Special Pathogens, Unit Biological Toxins (ZBS 3), WHO Collaborating Centre for Emerging Infections and Biological Threats, Robert Koch Institute, Seestrasse 10, 13353 Berlin, Germany

## Abstract

**Background:**

Over the course of the COVID-19 pandemic, laboratories worldwide have been facing an unprecedented increase in demand for PCR testing because of the high importance of diagnostics for prevention and control of virus spread. Moreover, testing demand has been varying considerably over time, depending on the epidemiological situation, rendering efficient resource allocation difficult. Here, we present a scalable workflow which we implemented in our laboratory to increase PCR testing capacity while maintaining high flexibility regarding the number of samples to be processed.

**Methods:**

We compared the performance of five automated extraction instruments, using dilutions of SARS-CoV-2 cell culture supernatant as well as clinical samples. To increase PCR throughput, we combined the two duplex PCR reactions of our previously published SARS-CoV-2 PCR assay into one quadruplex reaction and compared their limit of detection as well as their performance on the detection of low viral loads in clinical samples. Furthermore, we developed a sample pooling protocol with either two or four samples per pool, combined with a specifically adapted SARS-CoV-2 quadruplex PCR assay, and compared the diagnostic sensitivity of pooled testing and individual testing.

**Results:**

All tested automated extraction instruments yielded comparable results regarding the subsequent sensitivity of SARS-CoV-2 detection by PCR. While the limit of detection of the quadruplex SARS-CoV-2 PCR assay (E-Gene assay: 28.7 genome equivalents (ge)/reaction, orf1ab assay: 32.0 ge/reaction) was slightly higher than that of our previously published duplex PCR assays (E-Gene assay: 9.8 ge/reaction, orf1ab assay: 6.6 ge/reaction), the rate of correctly identified positive patient samples was comparable for both assays. Sample pooling with optimized downstream quadruplex PCR showed no loss in diagnostic sensitivity compared to individual testing.

**Conclusion:**

Specific adaptation of PCR assays can help overcome the potential loss of sensitivity due to higher levels of PCR multiplexing or sample dilution in pooled testing. Combining these adapted PCR assays with different sample processing strategies provides a simple and highly adjustable workflow for resource-efficient SARS-CoV-2 diagnostics. The presented principles can easily be adopted in a variety of laboratory settings as well as be adapted to pathogens other than SARS-CoV-2, making it feasible for any laboratory that conducts PCR diagnostics.

## Background

Testing for COVID-19 is an essential tool for managing patients, preventing and controlling virus spread, evaluating the epidemiological situation as well as guiding and monitoring public health protection measures. The reference standard for diagnosis of acute COVID-19 is the detection of SARS-CoV-2 RNA by nucleic acid amplification tests, usually real-time reverse transcription polymerase chain reaction (rRT-PCR). Antigen rapid diagnostic tests without confirmation by NAAT are only considered valid for COVID-19 diagnosis under certain circumstances [1, 2]. Between 30 December 2019 and 9 November 2022, the World Health Organization registered 630,387,858 confirmed cases of COVID-19 worldwide [3]. A total of about 6.8 billion COVID-19 tests has been performed globally since the beginning of the pandemic [4]. Consequently, an enormous strain has been placed on the laboratory infrastructure to scale up PCR testing capacities. Due to the high dynamics of the COVID-19 pandemic, including the occurrence of multiple waves, localized outbreaks and the emergence of several variants of concern [5], the demand for testing has been varying considerably over time, depending on the current global and local epidemiologic situation. SARS-CoV-2 RNA may be the analyte with the highest range in sample number per day or week that many laboratories have ever experienced. In Germany, the number of recorded SARS-CoV-2 PCR tests varied from about 500,000 to 2.5 million per week. The most dramatic change in the number of performed tests on the national level was seen at the end of 2021, when the number of performed tests per week doubled from about 1 million to 2 million within 5 weeks [6]. The number of SARS-CoV-2 PCR tests performed in our laboratory in 2020 and 2021 varied from about 200 to 4,900 per week and doubled or even tripled several times within 1–2 weeks.

As test results are usually required as promptly as possible, the ability to process a wide range of sample numbers in a time- and cost-efficient manner is generally desirable. Additional issues to be considered when scaling up PCR testing capacities include the average turn-around time, availability and affordability of reagents and consumables, available lab space and alternative options for equipment use if the increased demand for PCR testing is expected to be only temporary. We adopted a combination of measures to increase our PCR testing capacity while maintaining high flexibility regarding the number of samples to be processed. This included a nucleic acid extraction workflow allowing both single sample and 96-well plate-based extraction, a SARS-CoV-2 PCR assay including the option to apply two different levels of multiplexing and an easy-to-use sample pooling strategy.

## Methods

### Evaluation samples for comparing RNA extraction methods

RNA extraction methods were compared by using serial dilutions of inactivated SARS-CoV-2 cell culture supernatant and a panel of diluted SARS-CoV-2-positive patient samples. Supernatant was taken from cultures of the SARS-CoV-2 Munich strain after one passage on Vero cells and heat inactivated at 60 °C for 60 min. Complete inactivation was confirmed by three blind passages on Vero cells. Tenfold serial dilutions were prepared in PBS from the undiluted supernatant as well as from 1:2 and 1:4 pre-dilutions. One-time use aliquots were prepared and stored at − 80 °C until use. After preliminary testing, 11 dilutions around the limit of detection were chosen for performing the comparative analyses, with the highest concentrated sample having a Ct value of 30 in both the E-Gene and orf1ab rRT-PCR assay. To generate a panel of patient samples, 20 SARS-CoV-2-positive naso- and/or oro-pharyngeal swab samples were selected from routine diagnostics to represent a range of viral loads, with initially detected Ct values ranging from 16.8 to 35.6 in the E-Gene rRT-PCR and from 17.3 to 36.6 in the orf1ab rRT-PCR. Patient samples were thawed from storage at -80 °C, diluted 1:4 in PBS, aliquoted into one-time use aliquots and stored again at − 80 °C until use.

### RNA extraction and purification methods

Automated RNA extraction was conducted on the QIAcube Connect (Qiagen, Hilden, Germany), QIAcube HT (Qiagen), QIAsymphony SP (Qiagen), MagNA Pure 96 (Roche, Basel, Switzerland) or KingFisher Flex (Thermo Fisher Scientific, Waltham, MA, USA). The extraction instruments included in the study are commonly used and represent a range of different technical characteristics. All extractions were performed according to the manufacturers’ instructions. Carrier RNA was included in all extractions using Qiagen instruments. Kits and instrument protocols, lysis and inactivation procedures, sample volumes and elution volumes are listed in Table 1. In all extractions using the Qiagen instruments and the KingFisher Flex, 5 µl of virus-like particles containing RNA of an artificial sequence referred to as KoMa were included as internal control (IC). Extractions by the MagNA Pure 96 instrument were performed by using a feline calicivirus as IC.

**Table 1:** Details of RNA extraction and purification methods

Instru-ment (manufacturer)	Kit	Instrument protocol	Lysis and inactivation procedure	Sample volume (µl)	Elution volume (µl)	Conc. factor (sample vol. to elution vol.)
QIAcube Connect (Qiagen)	QIAamp Viral RNA Mini	QIAamp Viral RNA_Body fluid_Manual lysis custom-12^a^	560 µl of buffer AVL + 560 µl of ethanol (off-board)	140	60	2.3
QIAcube HT (Qiagen)	QIAamp 96 Viral RNA	QIAamp 96 Viral RNA OBL Protocol (high vacuum)^b^	560 µl of buffer AVL + 560 µl of ethanol (off-board)	140	80	1.75
QIAsym-phony SP (Qiagen)	QIAsymphony DSP Virus/Pathogen	ComplexOBL_2019-nCoV^a^	560 µl of buffer AVL + 560 µl of ethanol (off-board)	140	60	2.3
MagNA Pure 96 (Roche)	MagNA Pure 96 DNA and Viral NA Small Volume Kit	Viral NA Plasma ext lys SV 4.0	250 µl of external lysis buffer (off-board)	200	50	4
KingFisher Flex (Thermo Fisher Scientific)	MagMAX Viral/Pathogen Nucleic Acid Isolation	MVP_2Wash_200_Flex	0.25 mg of Proteinase K (on-board)	200	50	4

^a^Custom protocol: Sample lysis and addition of ethanol is performed manually

^b^Custom protocol: Sample lysis and addition of ethanol is performed manually. Maximum vacuum power is applied at all vacuum steps. TopElute Fluid is not used

### PCR analysis

The previously published version of the SARS-CoV-2 PCR assay was established as two duplex PCR reactions detecting E-Gene/KoMa-IC and orf1ab/c-myc, with c-myc serving as control for human nucleic acid content [7]. The assays are used with the AgPath-ID™ One-Step RT-PCR Reagents kit (Applied Biosystems, Foster City, CA, USA) on a Bio-Rad CFX96 Touch Real-Time PCR Detection System. Cycling conditions were set as follows: 45 °C for 15 min, 95 °C for 10 min followed by 45 cycles of 95 °C for 15 s and 60 °C for 30 s.

To establish a quadruplex version of the SARS-CoV-2 PCR assay, the fluorophore of the E-Gene probe was changed from FAM to HEX, and an additional probe was introduced to the orf1ab reaction to compensate for the observed loss in fluorescence intensity compared to the duplex assay. The primers and probes as used in the quadruplex assay are shown in Table 2. The final concentration of all primers and probes as well as the cycling conditions remained identical to those used for the duplex PCR assays. Table 3 shows the PCR mix composition of the quadruplex PCR reaction. Primers and probes were usually premixed and added to the master mix as a single component, i.e. as a primer/probes mix. The volume of RNA extract per reaction was increased from 5 µl in the duplex assays to 10 µl in the quadruplex assay.

**Table 2:** Primers and probes for quadruplex PCR reaction: E-Gene/KoMa/orf1ab/c-myc

Name	Sequence	O^a^	Position^b^	Tm^c^
E_Sarbeco_F1*	ACAggTACgTTAATAgTTAATAgCgT	S	26,302	53.9
E_Sarbeco_R2*	ATATTgCAgCAgTACgCACACA	A	26,414	57.9
E_Sarbeco_P1*	HEX-ACACTAgCCATCCTTACTgCgCTTCg-BHQ1	S	26,365	65.0
KoMa F	ggTgATgCCgCATTATTACTAgg	S	n/a^d^	57.8
KoMa R	ggTATTAgCAgTCgCAggCTT	A	n/a^d^	57.8
KoMa TM	TexRed-TTCTTgCTTgAggATCTgTCgTggATCg-BBQ	S	n/a^d^	67.7
orf1ab S	CTCTggAACACTTTTACAAgACTTC	S	19,594	54.5
orf1ab A	ACCATCAACTTTTgTgTAAACAgTg	A	19,731	56.3
orf1ab TMGB	FAM-ACAgggTgAAgTACCA-MGB	S	19,674	66.0
orf1ab add	FAM-TAATgTTgTAAATAAgggACAC-MGB	S	19,641	66.0
c-myc F	TAgTggAAAACCAgCAgCCT	S	380	57.0
c-myc R	TCgTCgCAgTAgAAATACgg	A	488	56.0
c-myc TM	Cy5-TATgACCTCgACTACgACTCggTgC-BBQ	S	442	63.5

^a^Orientation

^b^Position in GenBank entry (E_Sarbeco: Acc# NC_004718; orf 1ab: Acc# MN997409.1; c-myc NM_002467.6, GeneID: 4609)

^c^Thermodynamic melting temperature

^d^Artificial sequence

*Corman et al. [8]

**Table 3:** Master mix for quadruplex PCR reaction: E-Gene/KoMa/orf1ab/c-myc

Reagent	Vol. [µL)	Final concentration [nM]
Water	0.79	–
2 × RT-PCR buffer	12.50	–
nCoV E_Sarbeco F1 (100 µM)	0.10	400
nCoV E_Sarbeco R2 (100 µM)	0.10	400
E_Sarbeco P1 (100 µM)	0.05	200
KoMa F (100 µM)	0.075	300
KoMa R (100 µM)	0.075	300
KoMa TM (100 µM)	0.025	100
orf1ab S (100 µM)	0.075	300
orf1ab A (100 µM)	0.075	300
orf1ab TMGB (100 µM)	0.025	100
orf1ab add (100 µM)	0.025	100
c-myc F (100 µM)	0.03	120
c-myc R (100 µM)	0.03	120
c-myc TM (100 µM)	0.025	100
25 × RT-PCR Enzyme Mix	1.00	–
Mix Vol	15.00	
RNA Vol	10.00	
Total Vol	25.00	

The criteria for result interpretation in primary diagnostic testing of clinical samples remained identical to those previously described for the duplex assays [7]. In contrast to primary diagnostic, the PCR analyses performed in this study were interpreted in a simplified manner to allow comprehensive method description and comparison, i.e. all distinct amplification curves crossing the fluorescence threshold were considered positive, irrespective of their Ct value.

### Specificity testing

Due to the introduction of an additional probe to the orf1ab PCR in the SARS-CoV-2 quadruplex assay, we assessed the specificity of the quadruplex assay using SARS-CoV and MERS-CoV cell culture supernatant as well as 160 clinical specimens that had previously been tested positive for different respiratory pathogens (see results section for details). 74 of these clinical specimens had been tested with virus-specific assays using Ct value-based detection, showing Ct values between 19 and 24. The remaining 86 specimens had been tested using melting curve-based detection.

### Limit of detection

Probit analysis for the quadruplex PCR E-Gene/KoMa/orf1ab/c-myc was performed as previously described [7].

### Sample pooling

We applied a two-level pooling strategy with either two or four samples per pool. For both two-sample and four-sample pools, 100 µl of each sample were pipetted in a separate tube and thoroughly mixed by vortexing. The remainder of the original samples including the swabs was kept as retain sample for deconvolution of positive pools. 140 µl of the pool were used for subsequent lysis and RNA extraction by using the QIAcube Connect or QIAcube HT protocols (Table 1).

### PCR analysis of pooled samples

Pooled samples were analyzed by using a modified version of the SARS-CoV-2 quadruplex PCR assay, referred to as allFAM quadruplex assay, in which all probes for both E-Gene and orf1ab were FAM-labelled and thus detected in the same fluorescence channel. The allFAM quadruplex assay includes the additional probe for the orf1ab reaction, which is also present in the regular quadruplex assay, and also an additional probe for the E-Gene reaction. Table 4 shows the primers and probes as used in the allFAM quadruplex assay. Apart from adding the additional E-Gene probe with a final concentration of 200 nM, the master mix composition remained the same as in the standard quadruplex PCR (Table 3). Individual testing of samples from SARS-CoV-2-positive pools was performed by using the E-Gene/KoMa and orf1ab/c-myc duplex PCR assays, which enable the required two-target detection by measuring E-Gene and orf1ab signals in separate fluorescence channels.

**Table 4:** Primers and probes for all FAM quadruplex PCR reactions: E-Gene/KoMa/orf1ab/c-myc

Name	Sequence	O^a^	Position^b^	Tm^c^
E_Sarbeco_F1*	ACAggTACgTTAATAgTTAATAgCgT	S	26,141	53.9
E_Sarbeco_R2*	ATATTgCAgCAgTACgCACACA	A	26,253	57.9
E_Sarbeco_P1*	FAM-ACACTAgCCATCCTTACTgCgCTTCg-BHQ1	S	26,204	65.0
E TMGB add	FAM-CTTgCTTTCgTggTATT-MGB	S	26,171	67.0
KoMa F	ggTgATgCCgCATTATTACTAgg	S	n/a^d^	57.8
KoMa R	ggTATTAgCAgTCgCAggCTT	A	n/a^d^	57.8
KoMa TM	TexRed-TTCTTgCTTgAggATCTgTCgTggATCg-BBQ	S	n/a^d^	67.7
orf1ab S	CTCTggAACACTTTTACAAgACTTC	S	19,608	54.5
orf1ab A	ACCATCAACTTTTgTgTAAACAgTg	A	19,754	56.3
orf1ab TMGB	FAM-ACAgggTgAAgTACCA-MGB	S	19,688	66.0
orf1ab add	FAM-TAATgTTgTAAATAAgggACAC-MGB	S	19,641	66.0
c-myc F	TAgTggAAAACCAgCAgCCT	S	380	57.0
c-myc R	TCgTCgCAgTAgAAATACgg	A	488	56.0
c-myc TM	Cy5-TATgACCTCgACTACgACTCggTgC-BBQ	S	442	63.5

^a^Orientation

^b^Position in GenBank entry (E_Sarbeco: Acc# NC_004718; orf 1ab: Acc# MN997409.1; c-myc NM_002467.6, GeneID: 4609)

^c^Thermodynamic melting temperature

^d^Artificial sequence

*Corman et al. [8]

## Results

### Comparison of different automated RNA extraction methods

To evaluate the impact of the RNA extraction method on the sensitivity of SARS-CoV-2 detection, we compared five automated extraction methods by using serial dilutions of inactivated cell culture supernatant with SARS-CoV-2 concentrations around the limit of detection. PCR analysis was performed in duplicate by using the E-Gene/KoMa and orf1ab/c-myc duplex PCR assays, which resulted in a total of four PCR replicates for the detection of SARS-CoV-2 RNA in each sample. Table 5 shows the number of positive PCR reactions for each dilution when using the different extraction methods. Detection of SARS-CoV-2 from cell culture supernatant was comparable for all tested extraction automats. Results were confirmed by using two additional, independently generated series of dilutions (data not shown).

**Table 5:** PCR positivity comparison between five automated RNA extraction methods by using serial diluted cell culture supernatant

Sample	Dilution factor^a^	Q-Con	Q-HT	Q-Sym	KFF	MP96
1	–	4/4	4/4	4/4	4/4	4/4
2	1:2	4/4	4/4	4/4	4/4	4/4
3	1:4	4/4	4/4	4/4	4/4	2/2*
4	1:10	4/4	4/4	4/4	4/4	4/4
5	1:20	4/4	4/4	4/4	4/4	4/4
6	1:40	4/4	4/4	3/4	4/4	4/4
7	1:100	3/4	3/4	1/4	2/4	3/4
8	1:200	0/4	0/4	1/4	1/4	1/2*
9	1:400	0/4	1/4	1/4	0/4	1/4
10	1:1000	0/4	1/4	0/4	0/4	0/4
11	1:2000	0/4	0/4	1/4	0/4	0/4

PCR positivity comparison was performed by using serial diluted cell culture supernatant of SARS-CoV-2. The number of positive rRT-PCR replicates is shown for each sample. The SARS-CoV-2 rRT-PCR assay targets two regions of the SARS-CoV-2 genome and was performed in duplicate, yielding a total of four PCR replicates per RNA extract

Q-Con: QIAcube Connect, Q-HT: QIAcube HT, Q-Sym: QIAsymphony, KFF: King Fisher Flex, MP96: MagNa Pure 96

^a^The dilution factor relative to the most highly concentrated sample 1 is given in order to allow a relative comparison of performance around the limit of detection. In the pre-tests, sample 1 had a Ct value of 30 in both the E-Gene and orf1ab rRT-PCR assay

*RNA analysis not performed in duplicate due to insufficient volume of RNA extract

**Fig. 1:**
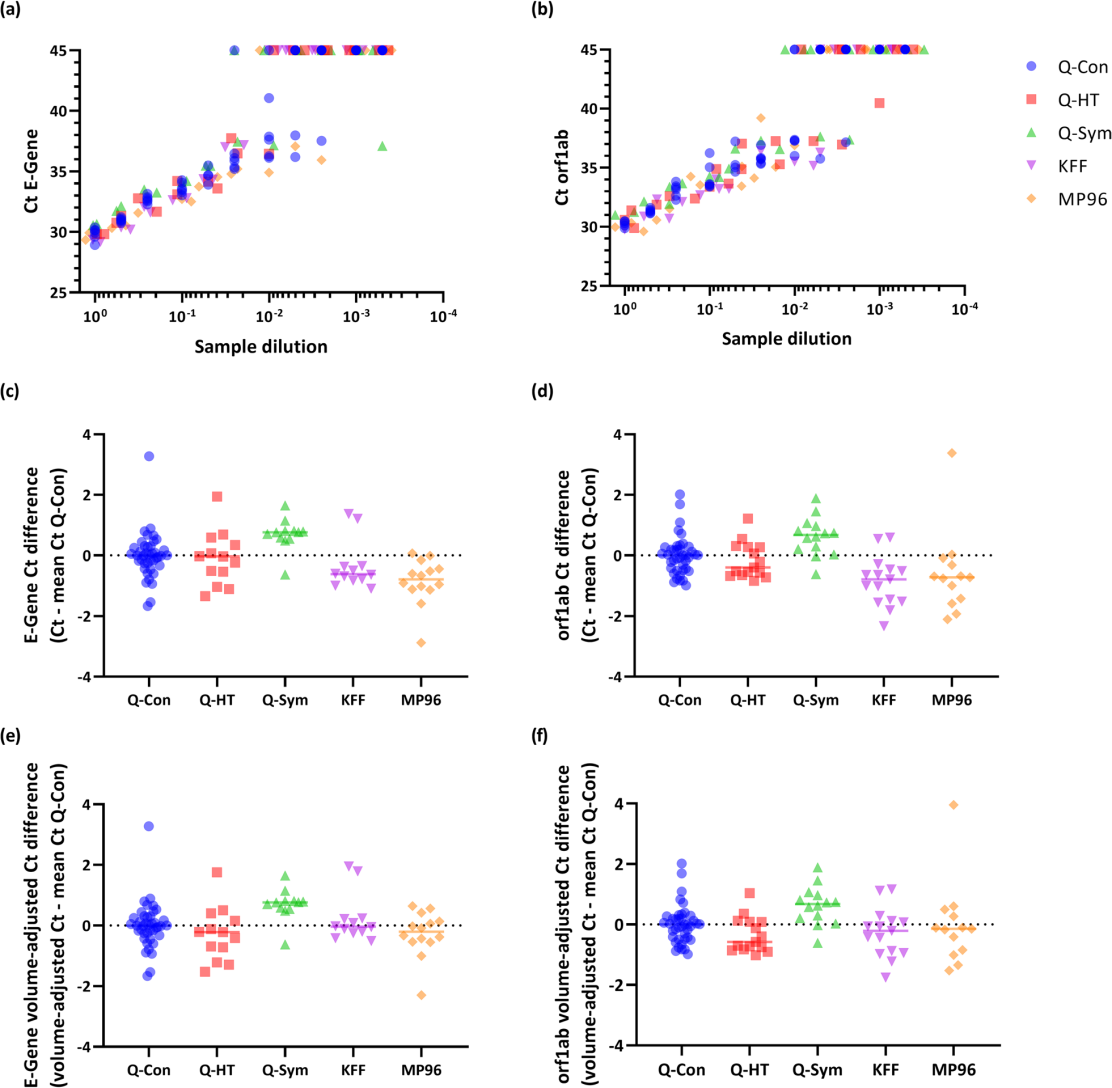
Ct values for detection of SARS-CoV-2 in serial diluted cell culture supernatant were compared between the five tested automated RNA extraction methods. **a** Ct values for each tested sample obtained with the E-Gene rRT-PCR and **b** orf1ab rRT-PCT. **c** Difference of the Ct value obtained for a sample extracted by the listed automat to the mean Ct value measured for the same sample after extraction by the QIAcube Connect in E-Gene rRT-PCR, **d** orf1ab rRT-PCT, **e** E-Gene rRT-PCR after adjustment for the ratio of sample volume to RNA volume and **f** orf1ab rRT-PCT after adjustment for the ratio of sample volume to RNA volume. Q-Con: QIAcube Connect, Q-HT: QIAcube HT, Q-Sym: QIAsymphony, KFF: King Fisher Flex, MP96: MagNa Pure 96

Figure 1a, b show the Ct values obtained for each sample in the E-Gene and orf1ab PCR after extraction by using the different instruments. Extraction using the QIAcube Connect was selected as reference method to quantify the differences in Ct values across instruments. For each instrument and sample, the difference of Ct values to the mean Ct value obtained with the QIAcube Connect was calculated (Fig. 1c, d). All automated extraction methods yielded similar Ct values in the SARS-CoV-2 rRT-PCRs. Using the King Fisher Flex and Magna Pure 96 resulted in slightly lower Ct values compared to the QIAcube Connect, which is probably due to the higher concentration factor of RNA extract in relation to the original sample (4 × concentrated when using the King Fisher Flex and Magna Pure 96; 2.3 × and 1.75 × concentrated when using the Qiagen instruments; Table 1). When the Ct values were adjusted for the different ratios of sample volume to RNA volume, the Ct difference of the King Fisher Flex and Magna Pure 96 compared to the QIAcube Connect was largely eliminated, illustrating the equal performance quality of the extraction automats (Fig. 1e, f). Using the QIAsymphony instrument resulted in slightly higher Ct values compared to all other tested methods, a fact which we also observed when applying different non-customized off-board and on-board lysis protocols provided by the manufacturer (data not shown).

The QIAcube Connect, QIAcube HT and QIAsymphony instruments, which at the time could be used for processing infectious samples, were additionally compared by using a panel of 20 SARS-CoV-2-positive patient samples, selected to cover a wide range of viral loads including virus concentrations around the limit of detection. All three compared extraction automats showed a similar performance on the tested patient samples, both with regard to the number of correctly identified samples and the obtained Ct values (Table 6).

**Table 6:** PCR positivity comparison between selected RNA extraction automats using 20 patient samples

Performance parameter	Q-Con	Q-HT	Q-Sym
Number of detected samples	20/20	18/20	18/20
E-Gene and orf1ab detected	16/20	15/20	14/20
Only E-Gene detected	3/20	0/20	3/20
Only orf1ab detected	1/20	3/20	1/20
E-Gene mean Ct value	30.76	28.92	30.56
E-Gene range of Ct values	18.46–38.72	18.21–40.08	19.20–38.20
orf1ab mean Ct value	30.80	30.95	30.47
orf1ab range of Ct values	19.31–37.42	19.10–38.46	19.94–37.10

PCR positivity was compared between three selected RNA extraction automats for detection of SARS-CoV-2 in 20 patient samples. PCR analysis was performed in duplicate by using the E-Gene/KoMa and orf1ab/c-myc duplex PCR assays.

Q-Con: QIAcube Connect, Q-HT: QIAcube HT, Q-Sym: QIAsymphony

### Increasing PCR analysis capacity through a higher level of multiplexing

Our SARS-CoV-2 PCR assay was initially developed as a combination of two duplex PCR reactions detecting E-Gene/KoMa and orf1ab/c-myc [7]. In order to be able to increase PCR throughput if needed, we combined the detection of E-Gene, KoMa, orf1ab and c-myc in a quadruplex reaction. As the fluorescence intensity of the orf1ab PCR suffered considerably from the higher degree of multiplexing, we added a second probe to the orf1ab reaction. Notably, this reduces the probability of detection failure due to virus mutations, for which the SARS-CoV-2-specific orf1ab PCR is at a higher risk than the more broadly reactive Sarbeco E-Gene PCR. For further compensation of the expected sensitivity loss in the quadruplex assay compared to the duplex assays, we increased the volume of RNA extract added to the PCR reaction from 5 µl to 10 µl.

The quadruplex assay showed no reactivity for patient samples that had been tested positive for influenza A virus (n = 22), influenza B virus (n = 2), parainfluenza virus 2 (n = 3), 3 (n = 4) and 4 (n = 1), respiratory syncytial virus (n = 24), human metapneumovirus (n = 20), rhinovirus (n = 98), adenovirus (n = 23), bocavirus (n = 8), NL63 (n = 29), 229E (n = 8), OC43 (n = 7) and Bordetella pertussis (n = 3). In addition, we detected no reactivity for MERS-CoV cell culture supernatant (n = 1) and only an E-Gene signal (as expected), but no orf1ab signal for SARS-CoV cell culture supernatant (n = 1). Probit analysis for the quadruplex PCR under the described reaction conditions (AgPath-ID PCR kit, BioRad CFX96) revealed a limit of detection of 28.7 genome copies per reaction for the E-Gene assay and 32.0 genome copies per reaction for the orf1ab assay. The limits of detection of the quadruplex PCR assay are therefore about three and five times higher than the previously determined limits of detection for the duplex PCR assays, which are 9.8 and 6.6 genome copies per reaction for the E-Gene/KoMa PCR and the orf1ab/c-myc PCR, respectively [7].

To evaluate the impact of the slightly reduced sensitivity of the quadruplex PCR on the ability to correctly identify positive patient samples, we compared the duplex and quadruplex PCRs by testing 24 patient samples that were previously identified to be SARS-CoV-2 positive with E-Gene Ct values higher than 30. Fresh RNA extracts were prepared from the original samples by using the QIAcube Connect and tested in parallel with the SARS-CoV-2 duplex PCRs and the quadruplex PCR. Both assays returned similar results for the tested samples (Table 7).

**Table 7:** Comparison of the duplex and quadruplex SARS-CoV-2 PCR assays using 24 weakly positive patient samples

Performance parameter	Duplex assay	Quadruplex assay
Number of detected samples	20/24	22/24
E-Gene and orf1ab detected	18/24	17/24
Only E-Gene detected	0/24	4/24
Only orf1ab detected	2/24	1/24
E-Gene mean Ct value	34.03	33.55
E-Gene range of Ct values	28.94–37.97	27.67–38.70
orf1ab mean Ct value	35.37	34.02
orf1ab range of Ct values	29.47–43.41	28.63–41.58

24 weakly positive patient samples were tested by using either the E-Gene/KoMa and orf1ab/c-myc duplex PCR assays or the E-Gene/KoMa/orf1ab/c-myc quadruplex assay

### Sample pooling

In order to prepare for peaks of exceptionally high testing demand, we established a two-level pooling protocol with either two or four samples per pool, which can be flexibly utilized depending on the current needs.

To minimize sensitivity loss caused by the dilution effect of the pooling, we developed a modified version of our SARS-CoV-2 quadruplex PCR assay, in which both E-Gene and orf1ab are detected by using the same fluorescence dye (FAM), which results in a higher fluorescence intensity. We refer to this assay as allFAM quadruplex PCR. While in the regular quadruplex assay an additional probe was included only for the orf1ab reaction, the allFAM quadruplex assay also includes an additional probe for the E-Gene reaction to further enhance fluorescence intensity and thus detection sensitivity. The allFAM quadruplex assay does not allow the distinction between E-Gene and orf1ab signals. However, each sample of a positive pool is subsequently analyzed individually by using the standard PCR assays which detect E-Gene and orf1ab separately. While successful RNA extraction and PCR inhibition can still be controlled for in the sample pools by using an exogenous internal control, confirming adequate sampling for each individual sample via an endogenous internal control is not possible when samples are pooled. Nevertheless, we decided to include the c-myc PCR in the assay for pooled samples in order to obtain at least some information on the human nucleic acid content in the pools.

We tested the performance of our sample pooling approach by utilizing the same 24 weakly positive patient samples that were used to compare the SARS-CoV-2 duplex PCRs with the quadruplex PCR (Table 7). In parallel to the individual testing, all 24 positive patient samples were analyzed in a pool with either one or three negative patient samples. RNA extractions were performed by using the QIAcube Connect, and all RNA extracts were subjected to the duplex PCRs, the standard quadruplex PCR and the allFAM quadruplex PCR. Compared to individual testing, both the duplex and quadruplex assays failed to detect three samples in the two-sample pools and six samples in the four-sample pools (Table 8). In contrast, the allFAM quadruplex assay did not miss any samples in the pooled testing compared to individual testing and even detected two additional samples in the two-sample pools.

**Table 8:** PCR positivity comparison between individual testing, two-sample pools and four-sample pools

Performance parameter	Duplex PCRs			Quadruplex PCR			AllFAM Quadruplex PCR		
	Indiv. testing	Pools of 2	Pools of 4	Indiv. testing	Pools of 2	Pools of 4	Indiv. testing	Pools of 2	Pools of 4
Number of detected samples	20/24	17/24	14/24	22/24	19/24	16/24	19/24	21/24	19/24
E-Gene and orf1ab detected	18/24	11/24	10/24	17/24	16/24	11/24	n.a	n.a	n.a
Only E-Gene detected	0/24	2/24	2/24	4/24	2/24	5/25	n.a	n.a	n.a
Only orf1ab detected	2/24	4/24	2/24	1/24	1/24	0/24	n.a	n.a	n.a
E-Gene mean Ct value	34.03	35.62	35.24	33.55	34.43	34.80	32.73*	35.11*	35.24*
E-Gene range of Ct values	28.94–37.97	28.44–41.69	29.19–40.04	27.67–38.70	27.06–39.69	27.99–39.26	27.81–36.28*	26.85–42.17*	27.97–41.69*
orf1ab mean Ct value	35.37	36.41	36.32	34.02	35.25	33.95	n.a	n.a	n.a
orf1ab range of Ct values	29.47–43.41	28.86–42.89	30.00–41.06	28.63–41.58	27.75–41.24	28.49–37.54	n.a	n.a	n.a

24 weakly positive patient samples were extracted individually and in a pool with either one or three negative patient samples. PCR analyses were performed by using the SARS-CoV-2 duplex PCRs, the standard quadruplex PCR and the pooling-specific allFAM quadruplex PCR

n.a.: not applicable

*The Ct values of the allFAM quadruplex PCR refer to the combined signals of the E-Gene and orf1ab assays as they are both detected in the same fluorescence channel in this assay

**Fig. 2:**
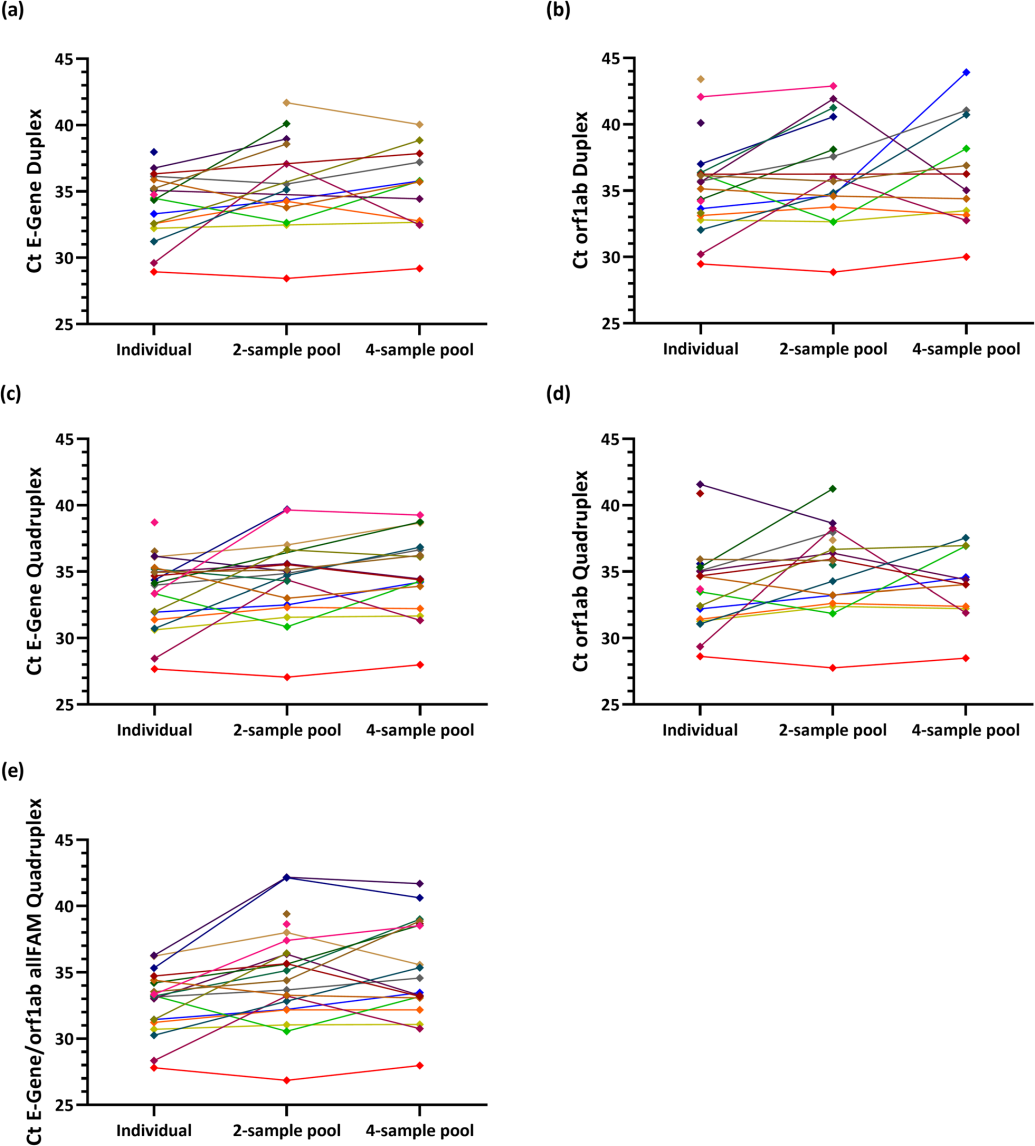
Comparison of Ct values between individual testing, two-sample pooling and four-sample pooling for detection of SARS-CoV-2 in 24 weakly positive patient samples. Shown are Ct values for each tested sample obtained with **a** the E-Gene PCR of the duplex assay, **b** the orf1ab PCR of the duplex assay, **c** the E-Gene PCR of the quadruplex PCR assay, **d** the orf1ab PCR of the quadruplex PCR assay and **e** the pooling-specific allFAM quadruplex PCR assay which detects E-Gene and orf1ab in the same fluorescence channel

Figure 2 shows the Ct values for each sample, obtained by individual testing, two-sample pooling and four-sample pooling using either the duplex assay (Fig. 2a, b), quadruplex assay (Fig. 2c, d) or allFAM quadruplex assay (Fig. 2e). Samples that were detected only by individual testing and not in the pools had Ct values of 34 or higher. While the average Ct values obtained from the pools were, as expected, about 1–2 cycles higher than those measured in the individual samples (Table 8), Fig. 2 shows that the difference in Ct values between individual testing and pooled testing varied considerably from sample to sample. In some cases, Ct values were even lower in the pool than in the individual sample.

## Discussion

When the demand for SARS-CoV-2 PCR testing started to rise in early 2020, we set out to increase our PCR testing capacity by about tenfold from roughly 100 to 1000 samples per day. The most challenging step was to increase the throughput of RNA extractions as several criteria had to be met. These included maintaining our existing workflow as much as possible, making optimal use of the available lab space, ensuring the availability and affordability of necessary reagents and consumables and maintaining the option to flexibly respond to changing workloads, both on a daily basis and with regard to the future ending of the pandemic situation. We compared five automated extraction methods and observed a similar performance of all tested extraction automats with regard to the sensitivity of SARS-CoV-2 detection. For routine diagnostics we decided to utilize an array of two types of compactly sized table-top instruments—the QIAcube Connect which processes 1–12 samples at a time and the 96-well plate-based QIAcube HT. Both machines can be operated with customized protocols allowing the identical manual lysis and inactivation procedure using 4 volumes of each buffer AVL and ethanol, which was demonstrated to inactivate SARS-CoV-2 with viral loads of up to 1 × 10^6^ PFU/ml [9]. The major advantage of this setup is its high flexibility. Extraction throughput can be adapted to the current demand without wasting reagents or consumables. The individual instruments can be arranged according to the available laboratory space, i.e. distributed across one or several rooms, and easily moved to other locations or re-designated to other purposes. The QIAamp viral RNA Mini kit that is used for the QIAcube Connect often serves as a reference kit, showing equal or superior performance in comparison to many other extraction kits [10–12]. It is also suitable for manual extraction which may be performed for example in the case of technical problems with the extraction machines. Its main limitation is the relatively high hands-on time due to the manual lysis and the need to operate several machines simultaneously.

**Fig. 3:**
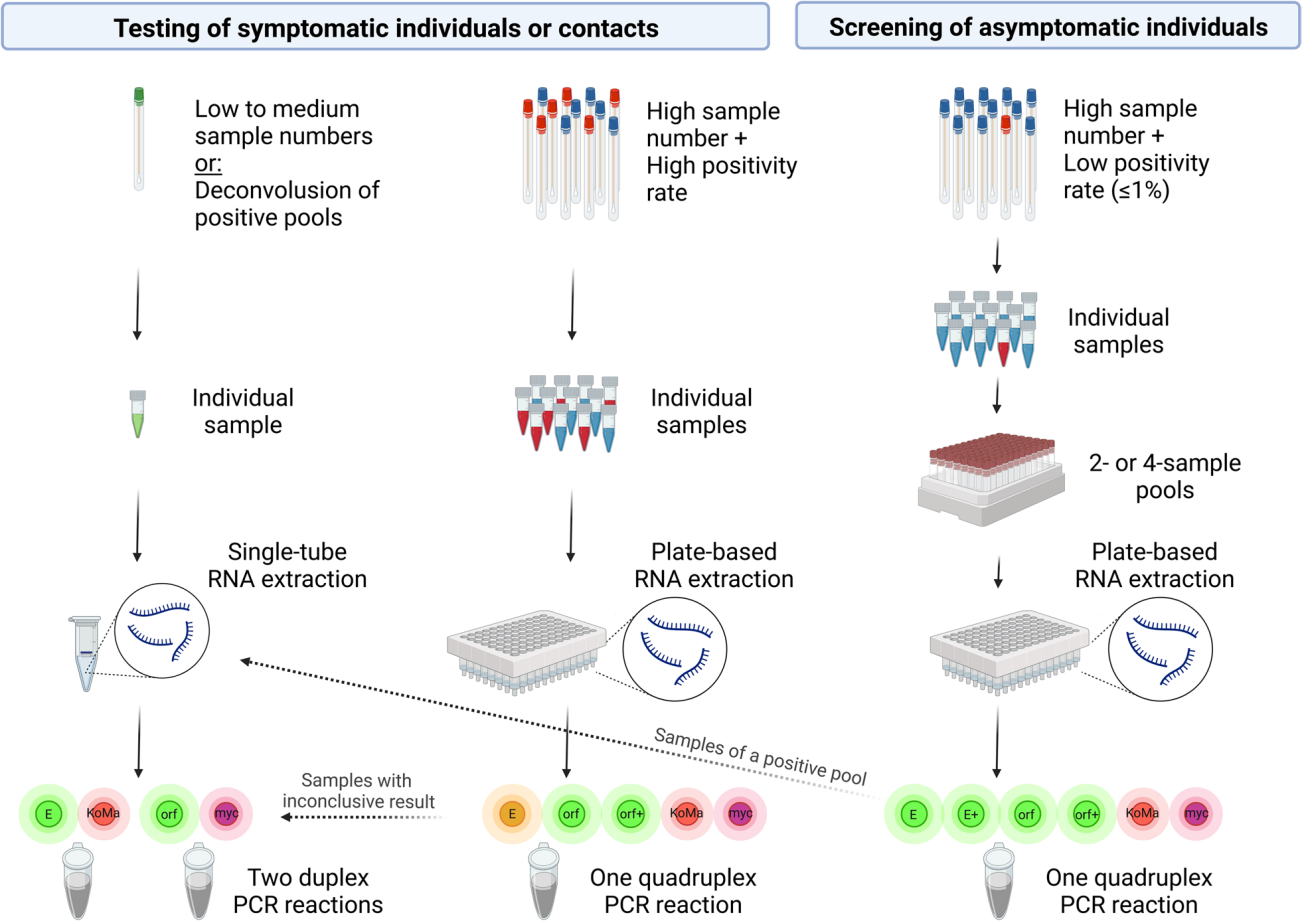
Overview of the different workflow options for SARS-CoV-2 PCR diagnostics in our laboratory

Increasing the level of multiplexing in our SARS-CoV-2 PCR results in reduced hands-on time for PCR pipetting and lower consumption of mastermix reagents and consumables per sample. It also enables the more efficient use of the available PCR cyclers. The main disadvantage is a slight, but detectable increase in the limit of detection compared to the duplex assays, even though the rate of correctly identified positive patient samples was comparable to that of the duplex assays in our tests (Table 7). Nevertheless, in order to minimize the risk of false negative results, samples that return an inconclusive result when using the quadruplex assay are retested using the duplex assays (Fig. 3).

Our sample pooling protocol enables flexible switching between individual and pooled testing during routine diagnostics, depending on the expected positivity rate and the number of samples to be processed. By adapting the downstream PCR analysis protocol, we were able to minimize the loss in sensitivity associated with sample pooling, thus ensuring comparability of results obtained from sample pools and individual samples.

Several different pooling strategies have been reported for SARS-CoV-2 testing, varying in pooling method and number of samples per pool [13, 14]. Pooling methods include pooling of swabs, either during collection or in the laboratory, pooling of swab transport media, pooling of saliva samples and pooling of RNA extracts [13, 15, 16]. We decided to pool swab transport media due to a number of practical considerations. This method can be applied to any type of swab, both dry and in transport media, and requires no special instructions or materials for sample collection. Because the remainder of the sample including the swab is kept as archive, retesting of individual samples is straightforward. While the pooling of swabs in a single container avoids dilution of each individual sample and therefore theoretically does not lower detection sensitivity, the retesting of individual samples poses a challenge in this approach. Collecting two swabs from each patient is a potential solution [17], but this is also resource-intensive and carries the risk of obtaining conflicting results from the two separate swabs. Swabs may be incubated in media individually to obtain an archive sample before pooling is performed [15]. However, this approach is probably influenced by the incubation technique and the swab’s characteristics with regard to releasing virus material into the media. Our own tests indicate a substantial drop in Ct values upon sequential incubation in media for all tested swab types (unpublished data).

**Fig. 4:**
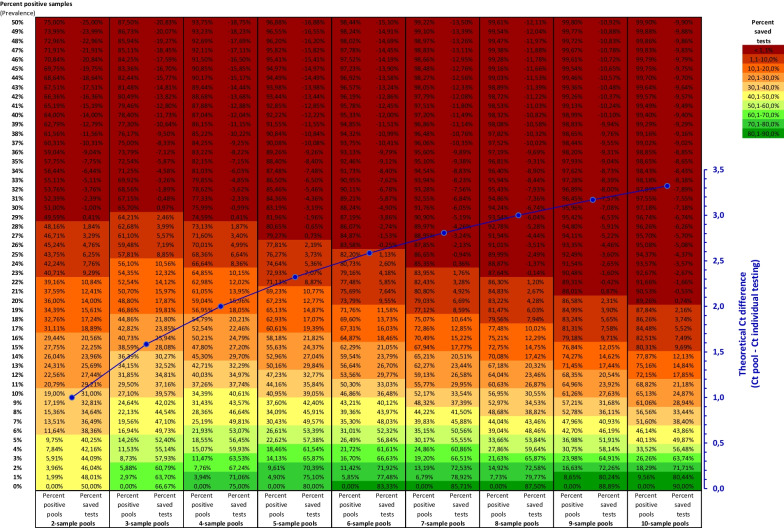
Overview of sample pooling parameters. Depending on the percentage of positive samples (prevalence), the mean percentage of positive pools and the mean percentage of saved tests compared to individual testing is listed for pool sizes of two to ten samples. The color code refers to the percentage of saved tests. The blue curve shows the theoretical increase of the Ct value compared to individual testing for each pool size $$\begin{aligned} {\text{Calculations}}: & {\text{Percentage of positive pools}} = 1 - (1 - {\text{prevalence}}){\mathrm{pool size}} \\ & {\text{Percentage of saved tests}} = (1 - {\text{prevalence}}){\text{pool size}} - 1/{\text{pool size}}. \\ \end{aligned}$$

The optimal number of samples per pool is primarily determined by the expected positivity rate, the acceptable degree of sensitivity reduction from sample dilution and the existing discrepancy between sample processing capacities and testing demand. The lower the positivity rate, i.e. the lower the prevalence of the disease in the tested population, the more tests can be saved by pooling. The samples we received for primary diagnosis were usually taken from symptomatic individuals and contacts of suspected or confirmed cases and had an average positivity rate of about 27%, which is not suitable for pool testing as the percentage of tests to be saved lies below 10%, even for a pool size of two [14]. We therefore applied pool testing only to screenings of asymptomatic individuals with high numbers of samples to be tested and an expected positivity rate of 1% or less (Fig. 3). While the optimal pool size for a prevalence of 1% is ten samples per pool, saving about 80% of tests [14], we decided to use pool sizes of two or four samples, depending on the total number of samples to be tested. At a prevalence of 1%, this still saves 48% and 71% of tests, respectively, while keeping the risk of handling and analysis errors to a minimum and limiting the theoretical shift in Ct values to 1 Ct for two-sample pools and 2 Cts for four-sample pools (Fig. 4).

By utilizing a pooling-specific version of our SARS-CoV-2 PCR assay which detects both viral targets with the same fluorophore, we were able to achieve a detection sensitivity similar to that reached with individual testing. In addition, using two or four samples per pool is also suitable for higher positivity rates, with 4 samples being the optimal pool size for prevalences of 7–12% [14] (Fig. 4), reducing the need to frequently adapt pool sizes to changing positivity rates.

## Conclusions

Faced with the challenge of the SARS-CoV-2 pandemic, laboratories worldwide have been applying a variety of strategies and methods to meet the exceptionally high testing demand [18, 19]. For molecular testing, these include rRT-PCR performed directly on crude samples without nucleic acid extraction [20–24], isothermal amplification techniques [25, 26] and CRISPR-based nucleic acid detection assays [27–29]. In our approach, we maintained a standard rRT-PCR testing workflow and the associated high detection sensitivity and specificity by combining three pragmatic and straightforward measures to stepwise increase our testing capacity, generating several options to flexibly adapt sample throughput according to the current demand.

## Data Availability

KoMa virus-like particles can be obtained on request.

## Abbreviations

Ct: Threshold cycle
ge: Genome equivalents
IC: Internal control
MGB: Minor Groove Binder
PCR: Polymerase chain reaction
rRT-PCR: Real-time reverse transcription polymerase chain reaction
